# Epidemic modeling with discrete-space scheduled walkers: extensions and research opportunities

**DOI:** 10.1186/1471-2458-9-S1-S14

**Published:** 2009-11-18

**Authors:** Maciej Borkowski, Blake W Podaima, Robert D McLeod

**Affiliations:** 1 Internet Innovation Centre (IIC), Electrical and Computer Engineering, University of Manitoba, R3T 5V6, Canada

## Abstract

**Background:**

This exploratory paper outlines an epidemic simulator built on an agent-based, data-driven model of the spread of a disease within an urban environment. An intent of the model is to provide insight into how a disease may reach a tipping point, spreading to an epidemic of uncontrollable proportions.

**Methods:**

As a complement to analytical methods, simulation is arguably an effective means of gaining a better understanding of system-level disease dynamics within a population and offers greater utility in its modeling capabilities. Our investigation is based on this conjecture, supported by data-driven models that are reasonable, realistic and practical, in an attempt to demonstrate their efficacy in studying system-wide epidemic phenomena. An agent-based model (ABM) offers considerable flexibility in extending the study of the phenomena before, during and after an outbreak or catastrophe.

**Results:**

An agent-based model was developed based on a paradigm of a 'discrete-space scheduled walker' (DSSW), modeling a medium-sized North American City of 650,000 discrete agents, built upon a conceptual framework of statistical reasoning (law of large numbers, statistical mechanics) as well as a correct-by-construction bias. The model addresses where, who, when and what elements, corresponding to network topography and agent characteristics, behaviours, and interactions upon that topography. The DSSW-ABM has an interface and associated scripts that allow for a variety of what-if scenarios modeling disease spread throughout the population, and for data to be collected and displayed via a web browser.

**Conclusion:**

This exploratory paper also presents several research opportunities for exploiting data sources of a non-obvious and disparate nature for the purposes of epidemic modeling. There is an increasing amount and variety of data that will continue to contribute to the accuracy of agent-based models and improve their utility in modeling disease spread. The model developed here is well suited to diseases where there is not a predisposition for contraction within the population. One of the advantages of agent-based modeling is the ability to set up a rare event and develop policy as to how one may mitigate damages arising from it.

## Introduction

Disease epidemic and pandemic modeling is an important and interesting area of study. There are widely held beliefs that a global pandemic or even a local disease epidemic in the general population is overdue. This notion is derived from time-series analysis of the occurrence of previous disease outbreaks, in which the time series includes a rare and high-impact event ('black swan' phenomenon) - an epidemic or catastrophe - that punctuates the equilibrium. This notion is also reflected in heavy-tailed distributions, which are inherently non-Gaussian in nature.

Our model is motivated by the ideals of a rational approach and inductive reasoning, augmented to encompass varying degrees of non-determinism and stochastic behavior, introduced as reasonable heuristics. Moreover, our model allows for expert input tailoring a disease phenotype in terms of modifying probabilities of contagions (that may infect); however, this too should yield to statistical methods.

Epidemic modeling is an important problem which garners a significant amount of public-health and public-policy attention, and thus warrants research not only from these fields, but from empirical and engineering perspectives as well. There are a large number of mathematical and simulation based approaches to epidemic modeling [e.g. [1-4]]. The majority of these mathematical approaches are quite dissimilar to the agent-based modeling presented here, in that ours is an agent-based computational model, essentially devoid of theoretically derived equations.

The basic tenets of our approach were the extraction of spatial (topologies) from maps, with extraction of agent behavior primarily derived from crude demographics and heuristics, resulting in the simulation of scheduled walkers on "correct by construction" topologies. The work is closely aligned with that of the Center on Social and Economic Dynamics [5] at the Brookings Institute and the many references contained therein. Organizations such as MIDAS [6] and PACER [7] also recognize the utility of large-scale efforts in agent-based computation and modeling benefits.

This paper is organized into two distinct parts. Part one is oriented towards our Discrete-Space Scheduled Walker (DSSW) simulation engine for epidemic modeling. This includes a section that outlines the basic underlying models and support for the conjectures made. Following that, we describe our implementation that captures the most important aspects of the specification. The last section of part one outlines the types of results that can be obtained. We conclude part one with a brief summary.

Part two highlights obvious shortcomings of the current DSSW simulation engine and outlines a number of data-mining opportunities that can be exploited for use within the DSSW framework; specifically, for extracting spatial and temporal patterns of behavior. This is followed by a brief overview of related work, illustrating the power and opportunities of data mining, from sources that are not necessarily related to epidemic modeling. Part two also includes a number of research opportunities and explores their feasibility in terms of the data sources required and data extraction availability. The last section outlines how the agent-based simulator could be used in assisting in the modeling of HIV/AIDS, albeit in a localized environment.

### The model

Data mining is a common theme in modern information technology. The basic tenets associated with data mining are:

1. Analytical methods may not exist or are overly complex.

2. Data does exist and can be readily extracted.

3. Statistical methods can now more easily deal with the vast amount of data that is available.

4. Oracles are giving way to statistical methods and number crunching.

Our work is an attempt to help serve and promote data-driven epidemic simulation and modeling. Where data is available, we demonstrate its utility; where unavailable, we demonstrate how it would be utilized. It should be noted that "unavailable data" often refers to the imposed practical or political limitations to information access, rather than to the technical or theoretical availability therein.

One of the key requirements for the implementation of the model under consideration is topological data, or physical "network" data - that is, *where *we are attempting to apply the study of epidemic spread. These are primarily real places, such as homes, institutions, businesses, industry, schools, hospitals and transportation hubs (and interconnects), within cities of all types. Until recently, the acquisition and procurement of such demographics would in itself be a daunting task. However, with the rapidly growing demand for "Google-like" mapping applications that rely heavily on data collection and dissemination, this type of data has become readily available. Hence, for the model under consideration here, this type of data repository is paramount for locating various institutions where people meet or come into contact with one another.

**Figure 1:**
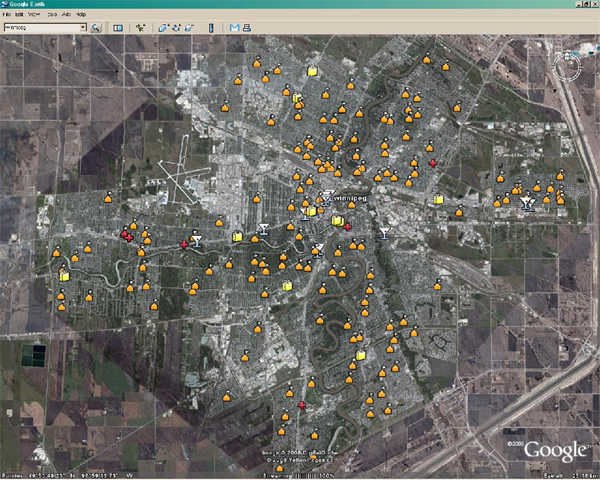
**Google map of Winnipeg with institutional location overlay**.

**Figure 2:**
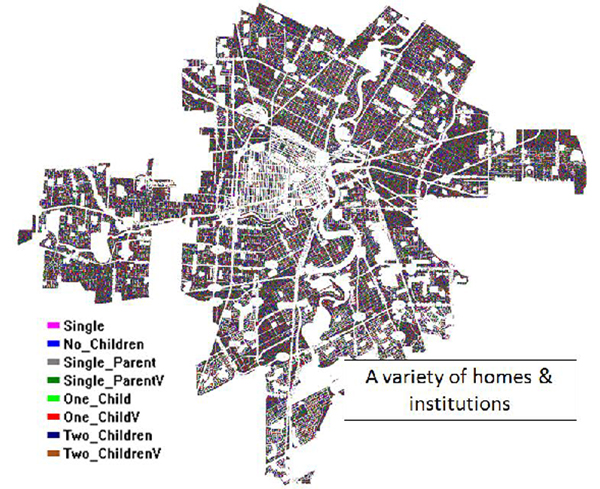
**Winnipeg topology with (simulated) institutions**.

For demonstration, the City of Winnipeg, Manitoba, Canada is used to illustrate the use of map information as input for generating our underlying topology of institutions where people (agents) potentially come into contact (interact) and infect one another. Figure 1 illustrates the City of Winnipeg's Google Earth map with various institutions identified (in yellow, orange, red), while Figure 2 illustrates the "corresponding" topological inputs for the simulator. Figure 2 can be edited and actual locations from Figure 1 embedded therein. In Figure 2, different colors represent different institutions; for example, a yellow pixel may represent homes with one parent and two vaccinated children.

In addition to incorporating institutional locations (*where*) in the model, other key components include the agents (*who*) themselves, who are being infected and capable of infecting. Knowledge of such data is generally technically available but practically unavailable. However, there are at least two approaches that could be used to obtain this data. The first is to collaborate with organizations that gather census data. Data mining these sources are not technical issues but rather political or policy issues and require the will of government and decision makers to make this data available for critical and credible applications (such as the simulator under consideration here). The second means of acquisition would be sampling a statistically significant percentage of the population, similar to an online data-collection portal, whereby information would be uploaded on a voluntary basis. Realistic collection of data from this latter source would likely be as difficult as mimicking an entire outbreak or epidemic in the first place. Additionally, significant effort would be required to design a voluntary data collection system that would capture truly representative unbiased samples of the various types of agents present within a total population. A present, our model attempts to illustrate how the data would be used, if available.

As well as *where *and *who*, agents' schedules (*when*) is also of critical importance. This data is typically inferred rather than explicitly available, but our conjecture is that we are primarily creatures of habit. Additionally, prior research supports the assumption that the majority of an agent's daily activities take place within a given radius of one's home and workplace (or school, etc.). It stands to reason that many of us operate on fairly routine weekday schedules, punctuated by more flexible weekends. As such, this scheduled data (for the sake of the simulation) can be associated with an agent, modified by slight variations in arrival and departure times. For example, a school-aged child spends the period between 8:00 AM and 4:00 PM at a local school with a reasonably high probability. Typically, they would be home at 4:00 PM, spending perhaps some of an evening for recreation (i.e., at a community centre or sports facility). The weekend schedule likely includes periods at home and periods locally within the neighborhood. While this profile retains a large degree of simplicity, it is a sufficient starting point in the representation of "schedule" in our simulation.

Each of these simulation aspects (*where, who, when) *can be extended to include a multitude of cities, weakly coupled by institutions such as planes, trains, automobiles and buses supporting agent movement. In addition, seasonal variation can also be incorporated, thereby accounting for variability in travel schedules and activities, and of course the seasonal prevalence of particular diseases (e.g., influenza).

In addition to the *where, who *and *when*, we also need to address the *what*. The *what *indicated here is typically a disease, either bacterial or viral in nature, communicated by physical human-to-human contact, and carrying a probability of infection when in contact with an infectious agent. These parameters are adjustable and represent an aspect of the current simulation with the greatest uncertainty in terms of their validity, and could benefit from collaboration or input from an epidemiologist.

### The implementation

The model above lends itself to an underlying simulation model we have denoted as a Discrete-Space Scheduled Walker (DSSW), in contrast to other models that are more traditionally based on random or Brownian walkers on irregular, computer-generated topologies. We attempt to capture the most important aspects of real-people networks, incorporating (correct by construction) notions such as "small world" or "scale free" networks.

**Figure 3:**
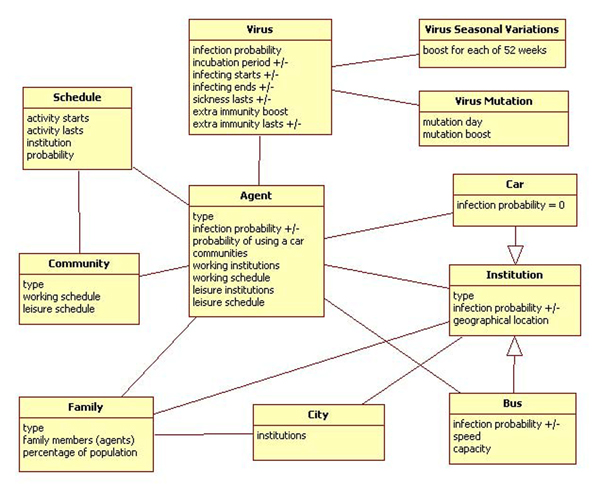
Object diagram for DSSW

Figure 3 shows an object diagram of the main objects used in the simulation (and their interaction). The following section describes how such objects are used in building the simulator.

### System overview

For illustration purposes here, the simulation area is limited to one city. A **City **is a geographical area where the simulation takes place. This area is defined by an image file incorporating institutions and homes, and is denoted by different colours in the input image (see Figure 2 for an example of a topology map simulating the City of Winnipeg). The central point of the simulation is an agent. An **Agent **represents a person within the simulation.

Another key component or building block of the simulation, which binds an agent with a physical place within a city, is an institution. An **Institution **is a place where an agent is allocated for a specified time interval. At all times during the simulation, an agent has to be assigned to an institution. There are three build-it special institutions: *Home*, *Bus *and a *Car*. A **Home **is a special institution, where agents start and finish their day. Homes perform two tasks. First, they are used to generate agents (see description of families). Second, homes are places where agents spend most of their time (including nights).

The mobility of agents within the city limits is implemented by buses and cars. Technically, buses and cars are institutions that are able to change their position. **Buses**, representing general public transit (e.g., subways, metros) as defined here, move always on straight lines from home of the first passenger to the destination institution. Buses are dynamically generated during the day to make it possible for all agents to reach their destinations. A bus is characterized by its probability of being infected, its capacity and its speed.

A **Car **is a special institution that simulates cars (and other forms of 'private' transit) in a city. The main characteristic of a car is that the infection probability inside a car is considered to be zero. The time spent in a car for commuting is equal to that spent in a bus.

Each day of an agent consists of activities. An **Activity **is defined by an institution where it takes place and the starting time and the period of time that it lasts. If an agent is not performing any scheduled activities, he/she stays at home. Using any means of transportation (eg., Bus or Car) is considered to be an activity as well.

All activities are organized into schedules. A **Schedule **is a list of an agent's activities along with the probabilities of choosing each activity. Schedules define what a given agent is going to do during the day. For each day, a new schedule is being generated. There are two types of days: **working days **and **leisure days**. There are also two types of schedules that can be defined for these days. A **Working schedule **is an agent's schedule used for a working day. A **Leisure schedule **is an agent's schedule used for a holiday. A **Working institution **is an institution where an agent works. The set of working institutions allows us to increase the number of places where a given agent (type) can work. When selecting the working institution for a given agent, institutions that are located closer to the home of a given agent have a slightly higher probability and therefore instance of selection. This probability can be adjusted by modifying the simulation parameters.

A leisure schedule defines activities that are assigned for given agent's leisure institutions. A **leisure institution **is an institution where agents spend their leisure time. This allows for the system to choose (in random manner) what agents do during weekends and holidays.

Working and leisure days correspond to weekdays and weekends/holidays, respectively. In addition, randomly generated weekdays are turned into holidays (to simulate public holidays or long weekends).

In order to simulate seasonal variation of infection rates, each week in a year has been assigned an extra parameter that defines the probability of being infected in a given week. For the infection probability formula, see equation [1].

Each day (24 hour period) is divided equally into timeslots. All activities can start or end only at a time slot. The time increment between timeslots is defined by an input parameter.

A population in a city is generated based on types and locations of families. A **Family **is a group of agents living in one home. Each family type can consist of any number of various agents. Each family has been assigned a percentage that sets the percentage of such family configurations (number and types of included agents) in the entire population of a given city. The actual number of agents in a city is a result of creating homes with families of a given type. Each home can be occupied by only one family. The geographical location of families within a city is random.

A **Community **is a "virtual" group of agents performing the same activities. Virtual means that no actual group is defined. A community is defined by working and leisure schedules. Each agent can belong to any number of communities. A schedule defined in such a community is added to the agent's schedule.

### Infection probability

The probability of being infected depends on several parameters given in Table 1. The infection probability formula is given in Equation (1).

**Table 1:** Daily Infection Probability Parameters

Variable	Meaning
*I*_*prob*_	probability of being infected
*i*	institution index
*j*	agent index
*d*	Day
*d*_*m*_	mutation day
*S*_*i*_	*i*^th ^institution
*T*_*i*_	time spent in *i*^th ^institution
Φ_*i*_	probability of being infected in *i*^th ^institution
Λ_*j*_	probability of being infected for *j*^th ^agent
*Ψ*	probability of being infected by a given virus
	probability of being infected in day *d*

Rather than considering equation (1) as a probability in axiomatic sense, the intent is to rationalize it as a factor that would contribute to the likelihood of infection. This likelihood of infection is more closely akin to Polya's calculus of plausibility as described in [8]. These random variables and their distributions can be refined if labeled data were available for comparison.

Factors such as  allow for experimentation with seasonal variation and mutation. Ideally, this would be correlated to historical seasonal variations or surveillance data where available. Mutation variation is experimental, adding flexibility to the model to simulate 'what-if' scenarios; for example, when viruses jump the species gap. This becomes a valuable utility for preparedness planning and modeling.

### Instantiation, scheduling and running

After all settings are read from configuration files, all instances of agents and institutions are created. First, institutions are created. A map of the city is searched and, depending on pixel colour, an institution of a given type is instantiated. The coordinates of newly created institutions are equivalent to coordinates of a pixel in an image. During instantiation, each institution is assigned an infection probability from a range of probabilities defined for a given type of institution. As a result, all institutions have slightly different infections probabilities. Only buses are created during runtime.

Once completed, simulation enters the phase in which agents perform their activities. First, the wall clock is set to 0:00. Then iteration through all timesteps begins. For each timestep, a list of activities scheduled for this time is iterated and agents are moved to their destination institutions. At this point buses are instantiated. Assume that an agent wants to travel from institution *S*_*i *_to *S*_*k*_:

• If an agent is scheduled to move to another institution right away, the agent is directed to move from *S*_*i *_to *S*_*k*_. Note: this situation happens when

- An agent is traveling in a bus or a car,

- An agent is within walking distance of the destination institution.

• An agent is removed from the source institution.

• A decision is made as to whether an agent uses a car.

- If an agent is using a car, the agent is moved to the car.

• If an agent is using a bus the following steps are performed.

- Based on a current location of an agent and all buses in a city, a bus that reaches the agent's destination (and is within a walking distance from an agent) is found.

- If such a bus exists, an agent is placed in this bus.

- Otherwise, a new bus is instantiated and an agent is placed in it.

• An activity, meaning that an agent is to get off the bus or a car, is added to the global list of activities (this activity is added to the timestep corresponding to the current time plus travel time).

After the above steps have been performed for all activities for a given timestep, the time is increased by an increment value and the processing of activities continues. The algorithm stops when the 24 hour limit is reached. After this point, the day counter is incremented and the next day is scheduled.

In general, running a day consists of moving agents from one institution to another. Because buses and cars are special types of institutions, using public transportation is already part of the process and does not require any extra steps. In addition, due to the event driven approach, there is no processing required for timesteps when no agent activity is present.

**Figure 4:**
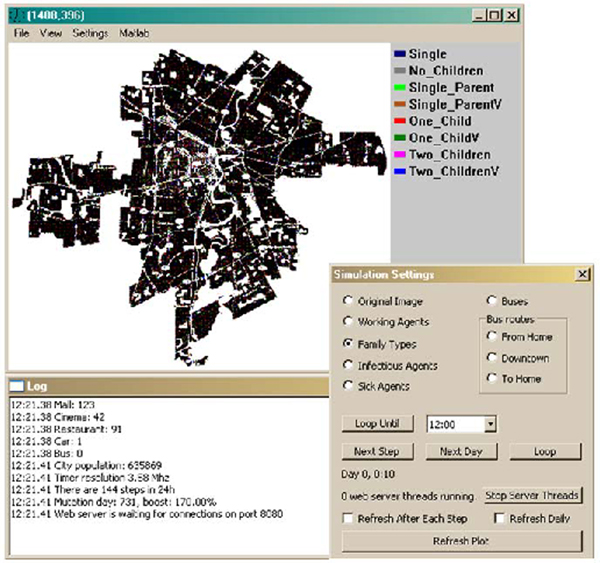
The user interface to DSSW

At present, the DSSW has an interface and associated scripts that allow for a variety of what-if scenarios to be demonstrated and for data to be collected on its server and displayed via a web browser. An example of this part of the user interface is illustrated in Figure 4. In this scenario, Winnipeg as an instantiation of a city is being modeled, using approximately 635,000 agents, the actual number of people in Winnipeg. In this example, the topology was extracted from Google Earth, whereas the population was simulated, varying the distribution of family units and types across the city. In this particular example, we were able to simulate the effect of various vaccination or antiviral policies similar to those currently being proposed by the CDC (recommendations of flu vaccination for children from ages 6 m-18 y, in contrast to previous policy of vaccinating children up to 6 years of age [9]). Parameters for simulation are set up in a number of files and the user can step or loop through the simulation at any given rate. During the simulation, a number of plots and statistics are collected and logged to a web server where the user can then further analyze the simulation run.

**Figure 5:**
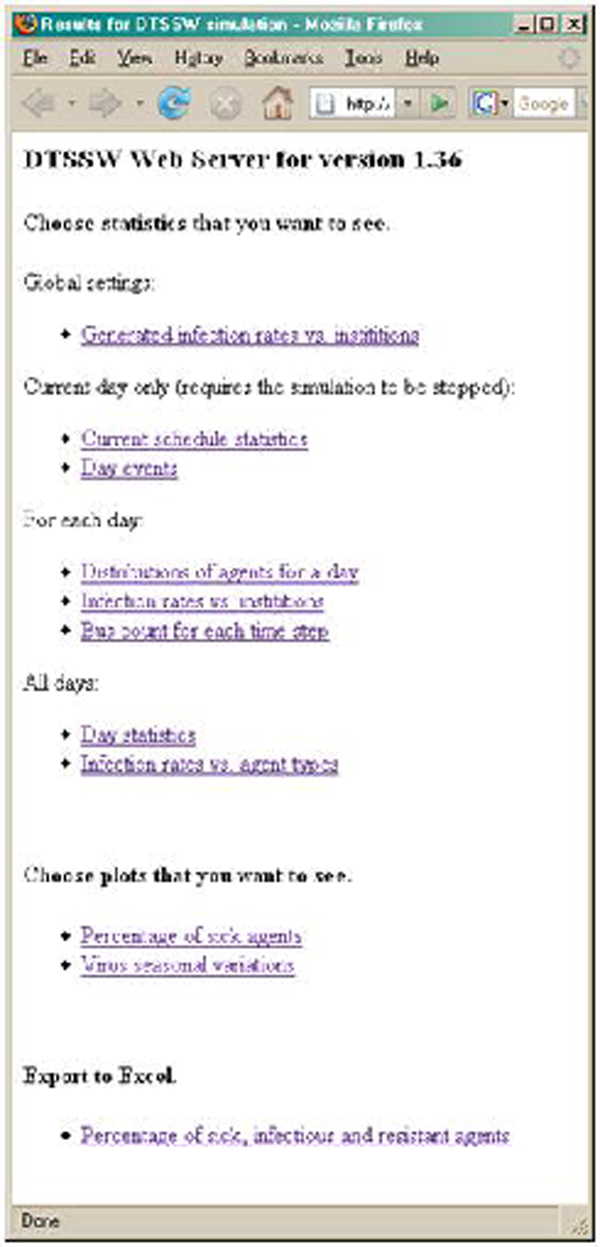
**Web Server Statistics for DSSW**.

**Figure 6:**
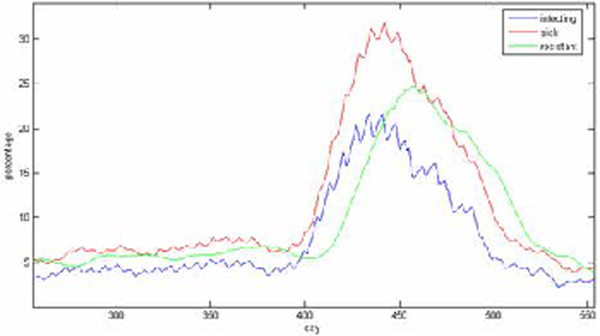
**The Simulation Augmented with a Seasonal Variation**.

**Figure 7:**
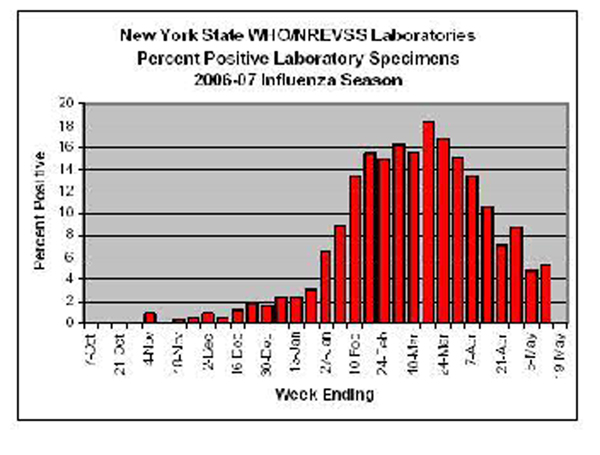
Labeled influenza seasonal variations

Figure 5 illustrates some of the data that is available on the corresponding web server. Figure 6 illustrates a simulation with a superimposed seasonal variation. Seasonal variations are well known and provide fairly well-labeled data for comparison. Figure 7 illustrates the type of data available that would be suitable for comparison to the simulated results and allow for a tuning of parameters to more closely reflect actual data collected concerning a particular disease [10]. Seasonal variations for Winnipeg can be directly inferred or mined from WRHA [11]. It would be desirable to automate the mining from web sites such as these, perhaps using data-mining tools such as [12].

**Figure 8:**
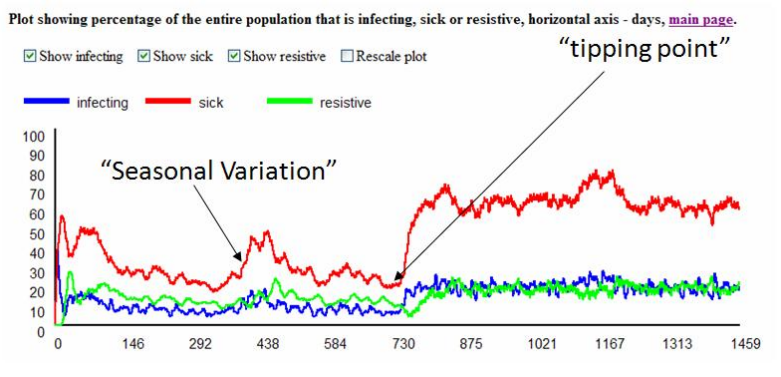
Virus mutation scenario

The simulator also allows for some degree of control, such that "what-if" scenarios may be undertaken. For example, during a simulation, at Day X, we may wish to infer the severity of the virus changing by means of a mutation into a potentially deadlier strain or a sudden variation in the mode of transmission (perhaps the virus has become airborne). Other potential benefits of the simulator include helping to evaluate to what extent methods of inoculations or epidemiological management policies (e.g., restrictions on movement) have in the circumvention of an outbreak event. This will allow for epidemiologists to partially "close the loop" when evaluating policy. Using extensions such as those discussed above, a simulation can be run emulating a mutation. Figure 8 clearly illustrates this type of situation.

This observation brings up another aspect for consideration. In addition to disease spread and modeling, we might be able to model the effectiveness of timely vaccination and/or inoculations. Typically, vaccination clinics are more likely be held at relatively large institutions throughout a city (school, community centre, etc.) or through other programs offered through the workplace or regional health authorities. These services would have considerable consequences in perhaps limiting the rate at which disease could spread and would also indicate potential bottlenecks in delivery, particularly if the vaccine were of limited supply. It does, however, open potential avenues of modeling, such as the Prometheus effect.

Also potentially having an effect on the spread of disease and the modeling therein are the wide spread usage of anti-viral and anti-retroviral medications. Some anti-viral medications are specifically designed to mitigate the effects of influenza and the associated symptoms, and reduce the amount of time an individual may be infectious. Some are known to have an effect on type 'A' and type 'B' influenza-causing viruses. Tamiflu and Relenza are two such medications that belong to a separate class of medicines called neuraminidase inhibitors [13]. They work by stopping the particles of the virus from being released into human cells, hence limiting the *in vivo *spread of the infection. It is not know precisely how effective these kinds of anti-viral medications really are, but nevertheless, with widespread adoption, it is likely that models of disease spread will have to be adjusted accordingly to incorporate their effects.

These are some of the kinds of simulation studies that are made possible by taking data that is fairly readily available and augmenting it by rational behavior patterns of people-people interactions. We are in the process of making the DSSW project for epidemic modeling an open source application; we will be initially hosting and supporting it on the Internet Innovation Centre web site http://www.iic.umanitoba.ca, and subsequently porting it to source forge when more stable.

### DSSW summary

This section introduces a rational method of epidemic modeling that takes advantage of opportunities for data mining of readily available data and what we call Discrete-Space Scheduled Walkers. The basic characteristic and contribution of the model is to extract and combine real topographic and demographic data. While previous models often rely on artificial data, this work shows that model creation using real data is indeed feasible and will likely result in a better characterization of the actual dynamics of an epidemic outbreak. Further work will focus on refining the model and validating the aforementioned conjectures.

Our goal in this section was to simply present a rationally motivated and inductive approach that, if successful and deemed of some utility, will necessarily involve the clinical expertise of practitioners and others with interest in preparedness planning or mitigation of damages.

### Extensions and opportunities

This section and the next (effectively part two of the paper) outlines limitations of the current DSSW model and presents a number of research opportunities for others interested in epidemic modeling and data mining..

### Current limitations of the DSSW epidemic simulator

Of course, our model is preliminary and simplistic, and experts in the field will be able to point out the subtle as well as not so subtle nuances. At this point, it may be worthwhile to acknowledge some of the simplicities/limitations/idiosyncrasies, such as the following:

We only allow for two types of activities: work and leisure. Many people's lives are not so clear-cut. Many people have two paid jobs. Some people may spend their time volunteering at an unpaid job, such as caring for an aging parent and "leisure". The whole unpaid work economy is not well represented in this model, because it is often not associated with a single formal institution, but is nonetheless significant in the movements and activities of a population.

Second, the current model schedules as weekdays and weekends. Although most people in the paid workforce do have two days off during the seven-day week, they do not all overlap or else none of us would be getting our groceries or gas on the weekend. The model does not currently accommodates this, with everyone in the model having leisure schedules on the same days. This is not a technological difficulty, as individuals could be incorporated that have more general schedules.

The geographic location of a given family type (number and types of agents) within a City is random - however, this is obviously not true. Certain areas will support larger family types (e.g. correlating low-income areas with larger family sizes) and different family configurations (e.g. families consisting primarily of adults in areas known for student housing, e.g. Wolseley or granola-type areas of Winnipeg). Census data will be able to refine this.

Also, at present, in the defining of a car, the infection probability is considered to be zero. This statement assumes a single rider - and discounts the soccer mom phenomenon - again indirectly related to the unpaid work economy as well as leisure schedules.

Having acknowledged some of these more obvious limitations, the next sub-sections lend themselves to a discussion of more extensive modifications and data-mining opportunities. Our emphasis is on data mining of topological structures (networks) and the utilization of patterns of behavior as the cornerstones of the simulation effort. The following will also outline data-mining opportunities of an often less-than-obvious nature that may have a significant effect on epidemic modeling.

### Hierarchy

The DSSW simulator is based on patterns of spatial and temporal behavior within a city. This can be generalized to multiple cities as mentioned herein. In addition, although not mentioned, hierarchy can be introduced. Cities can form a hierarchy within provinces (states/regions) and those within a hierarchy of countries, continents, etc. The basic modality remains: data-driven models of Discrete-space- and time-walkers, mined from available sources. In a multiple city simulation, transportation becomes an extremely important factor in hierarchy but also allows for the problem to remain tractable and potentially allow for efficient modes of computation if parallelism is exploited. That is, models for cities are primarily autonomous, exchanging information at borders or gateways. This is a technological issue and not a theoretical obstacle to the modeling. As such, two informal hierarchical extensions to DSSW are somewhat obvious, the one mentioned above as well as several layers of hierarchy, perhaps within large institutions (universities, schools and malls). It is likely, however, that the level of hierarchy would remain quite shallow.

A specific research opportunity lies in refining a model for a particular institution such as a University, dealing with all parameters thoroughly and to a high degree of accuracy, based on explicit assumptions and a high degree of sensitivity. In this specific instance, we will attempt to mine the University of Manitoba's student database system for agent behaviour, demonstrating the utility of exploiting Institutional Hierarchy. Whether this level of detail is required or not is an open question. The work here would be on using agent-based models as support for knowledge bases which can then be further developed to perhaps provide simple rules-of-thumb institutional models, providing a demarcation between the details required and perhaps where computationally simpler models could take over. Emerging technologies such as WiFi tracking [14] could be used to provide statistically significant behavioral-movement patterns and serve as empirical inputs to the simulator. These technologies are being developed to enhance campus security but could easily be extended to provide a better model of agent mobility and hence disease spread.

Another specific research opportunity is an *EPI@home *project. This is a very exciting research opportunity and will serve as a means of extending the DSSW simulator to model interactions among a variety of cities with any number of participants. In its initial manifestation, it is really a cluster-based implementation of our agent-based epidemic model. In a cluster, each node would run an instance of a city and communication would emulate people traveling via air or train. Our packets in the TCP/IP sense would transport agents as their payload. We have started an EPI@home Google site and have just taken epi-at-home.com as a starting point to further disseminate the research which will be an open source grass roots initiative.

### Extracting use-case and patterns of behaviour

Means of extracting patterns of behavior could be taken from tracking technologies that are already in place - albeit not mined or exploited to the best of our knowledge - for generating behaviors that could be used in epidemic modeling.

These include financial transactions and cell-phone location technologies. Briefly, financial transaction tracking is widely deployed as a means of detecting fraud where anomalous behavior is flagged when unusual purchase-behavior patterns are inferred. The techniques used are usually related to profiling and learning customer behavior and monitoring events to uncover indicators of anomalous or fraudulent behavior from a database. Profile behavior information would provide additional empirical input to the DSSW simulator when combined with other demographical data where available. Of course, this would again be statistical in nature, providing a sample of patterns that could be used to augment modeled behaviors. The information mined, however, would not be the more difficult problem of determining anomalies, but rather the behavior profile itself. Most people, however, are particularly uncomfortable with this level of intrusion (more to do with perception than anything concrete) and data of this type are likely difficult to obtain without the involvement of the financial institutions themselves. Bio-surveillance centers do monitor for financial transactions such as prescription and over-the-counter medication (e.g., anti-diarrheals, anti-nauseals) purchases at pharmacies that are used as inputs to analysis tools that attempt to predict the potential outbreak of a disease. To some degree, this is already being done, whether people are aware or not.

The cell-phone industry has long been aware of "where" mobiles are, such that they can route calls as users move within a city or region. In addition, a number of new features are being added that enhance cellular location based services. These include E-911 as well as "where are you" services. Many newer cell phones will be or are already equipped with GPS, that allow greater location accuracy. In some cases, these technologies will and are being used in conjunction with the telecom service provider to deliver location-based services. For our purposes, only a statistical sample is required, as behavior patterns of others can be modeled around these samplings of baseline behaviors. In an extended services capacity, cell-phone tracking data would provide input to where people are and their patterns of movement. Clearly, this information exists (and is incorporated in some telecom client services) but would not likely be publicly available on a mass populous scale and certainly not unless sanitized. There are hash functions that can provide electronic "fingerprints" of users without actually revealing the identity of the user if this type of data were to become available. We plan on emulating this type of behavior-capture within the DSSW simulator. Substantiating our claim that this type of data may become available, it is worthwhile to note that, in addition to consumer-location-based services, there are a growing number of patents whereby data from cellular providers would augment a city's traffic light control system with dynamic traffic information. Clearly, if these systems are to be employed, issues such as privacy (Privacy Legislation) will be of paramount importance.

Three specific research opportunities present themselves in this fashion.

**Figure 9:**
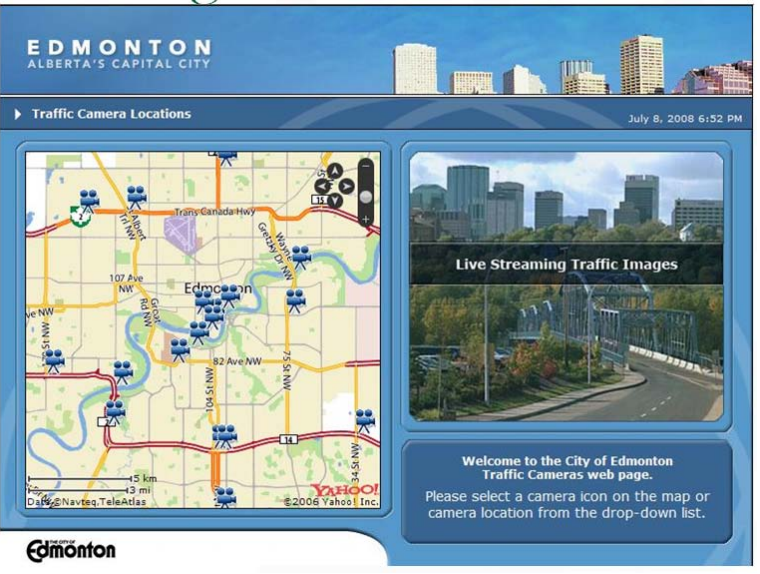
**City ITS traffic cams **[15]

**Figure 10:**
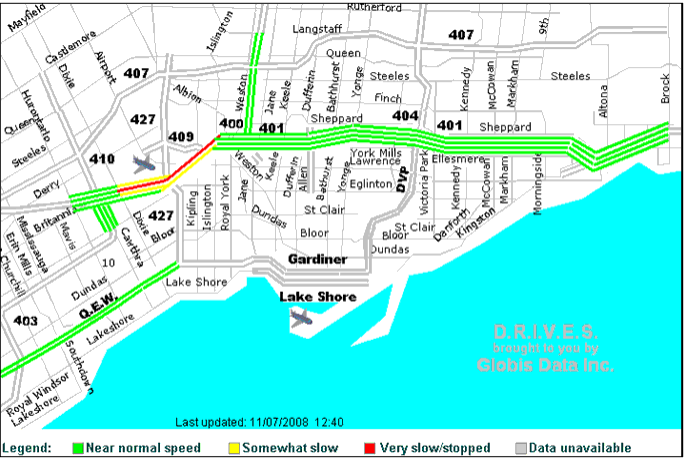
**Toronto "Real Time" traffic **[16]

**Figure 11:**
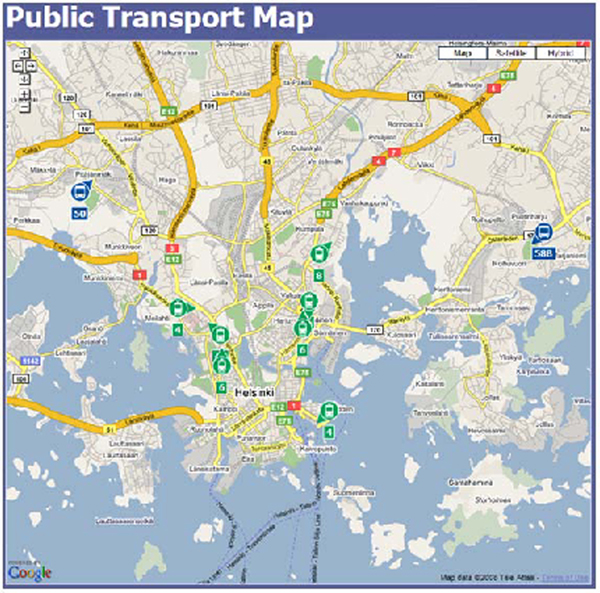
**Real-time public bus transport map **[19]

1) Model a "major" city such as Toronto with the Greater Toronto Area population of approximately 3.5 million people. In Toronto and similar large cities, the dynamics are considerably different than that of medium-sized cities like Winnipeg. In Toronto, there is a great deal of arterial flow of large numbers of persons. In this regard, it is worthwhile to model the behaviour of traffic flows into and out of Toronto as a means of modeling how the spread of a disease may occur between localities. Many cities have webcam traffic monitoring (publicly available) from which flows can be inferred. Image-processing tools (e.g. using OpenCV) will be required to demonstrate the utility of extracting traffic flow and illustrate the utility of traffic flow inferencing in epidemic modeling. In the long term, real-time satellite views may become available which we would then use. At this year's Intelligent Transportation Systems Congress, not one explicit link between intelligent transportation systems and epidemic modeling was evident; illustrating the tremendous opportunity we have in this endeavor to address an underexplored area of epidemic research. Figure 9 (albeit Edmonton) and Figure 10 illustrate emerging sources of data extraction ripe for use as empirical inputs into the DSSW model.

2) Model a "strange" city like Las Vegas, where 80 million visitors a year arrive and stay for three to four days at a time. Las Vegas is a "destination city" (as opposed to other major cities, where a similar number of people may arrive, but immediately transit through to other destinations). Thus, Las Vegas offers a different kind of opportunity for a disease to propagate than a majority of cities. It is possible to mine air traffic schedules to get a very reasonable flow in and out of Las Vegas. There are a defined number of hotels, all mineable from Google Maps or equivalent. In addition, a significant percentage of persons in Las Vegas work in the service industry. The most promising airline mining being considered as a first phase will be from add-ons such as those at Yapta's [17] or Flightaware's [18] flight-tracking website Although initially designed for providing a consumer with flight information, these can also be mined and provide empirical data for an epidemic simulator.

3) In any city, mining behaviour through scheduled public transport is a good way to model contracting a disease. Public transit systems transport people in high volume and high densities. Estimating ridership would likely be a challenge, but could be made available if the efficacy of the modeling could be demonstrated. An immediate goal of the DSSW for the Winnipeg model is to incorporate public transit (bus) route and schedule data to refine the relatively simple public transportation model currently employed. Extended research opportunities will come from applications such as the real-time tracking of bus corridors and/or subway systems. The system referenced in [19] uses actual transportation real-time maps to indicate the movement of busses within a city. The city illustrated is Helsinki and they monitor the bus transportation system using GPS, a display of which is illustrated in Figure 11.

Their objective was to provide information for riders; however, our interests would be using this data to model the movement of people within a city for disease modeling and its possible spread thereof through their behavior patterns. The idea purported here is that we would mine sites like these to improve the DSSW within a city if the data is available. Within DSSW, these inputs would provide a better estimate of the potential of contracting a disease within a public-transportation system (an object within DSSW). Asset-tracking technologies such as those based on WiFi are also being developed as controls for public transport as complements to GPS [20]. These types of integrated emerging technologies and applications could provide incredible data resolution for an epidemic simulator.

### Extraction of data from less-obvious sources

The following discusses an indirectly related reference [21] that illustrates the use of 'Bluetooth' to track travel time for vehicles and pedestrians. It is used here to illustrate that these technologies are being developed and could be adapted for use as data inputs to a DSSW epidemic simulation.

In [21], it is reported that, "Travel time is one of the most intuitive and widely understood performance measures associated with transportation services. However, it is also one of the most difficult performance measures to accurately estimate. Toll tag tracking has demonstrated the utility of tracking electronic fingerprints to estimate link travel time. However, these devices have a small penetration outside of areas served by toll facilities and the proprietary tag reading equipment is not widely available." Their paper [21] reports on tracking of a wide variety of consumer electronics that already contain unique digital fingerprints. These fingerprints are often associated with the physical addresses (e.g. MAC) needed when devices communicate. These MAC addresses are by necessity in plain sight and tend not to change over time. In their study or proposal, consumer electronic devices would be "snooped", allowing for a statistical estimate of ingress and egress of arterials. Travel time data would then be presented for both freeway and signalized arterials. Expected travel times and their variance would graphically illustrate variances associated with weather and traffic conditions. The paper concludes by discussing privacy concerns and recommending several applications in the area of arterial performance measures, construction work zone travel time and travel time across various transportation modes. An obvious extension would be in providing patterns of behavior of a representative percentage of the population (i.e., a statistically significant ensemble of people) and could easily be extended to public-transportation systems. It is reasonable to assume that more widely available WiFi, and WiFi-enabled devices such as smart telephones and PDAs, could also be used in a similar manner as much of the same types of low-level wireless protocols are standardized.

Furthermore, publicly available and electronic (wired and wireless) device-enabled Kiosks and vending machines are becoming more mainstream and are being incorporated over a wide regional demographic. These may also help serve in contributing to the acquisition of representative patterns of behaviours (location and tracking).

Also widely disseminated and integrated among the public are security cameras and systems with automated person detection (and face recognition/identification). Many of these systems have been installed for the purpose of monitoring for behaviour patterns more conducive to illegal activities and terrorist threats. They are increasingly being used for security and surveillance in urban centers - for locating and tracking, normally of parties of interest by authorities - and can likewise be used for the determination of public behaviour patterns in a similar capacity. Because there are many such systems already deployed worldwide in the field (e.g., streets, traffic/pedestrian lights, transit corridors and hubs), it makes perfect sense to make use of this data for epidemiology modeling and the like. Of course, the utility of this method is also fraught with the same concerns over privacy law, and data availability and accessibility.

Reference [22] has a short section on data mining subway systems' information (basically ridership) for patterns of busy behavior, number of people on the subway, etc. Some of this work is being done to provide behavior patterns that would be a factor in a bioterrorism attack and its impact. However, it is the mention of the "mining" of this type of information that is of interest to us here. The notion that this information may have been collected for purposes such as actual scheduling but may be extendable for use in a simulator as behavior pattern inputs is encouraging.

Although not as explicit and readily attainable, the potential to extract patterns of behavior and interactions of agents at critical institutions such as hospitals may be made feasible through the use of Radio Frequency IDentification (RFID) tracking. As RFID sensor networks move from inventory solutions to enhanced applications, data collected from RFID tracking at clinics and hospitals may be envisioned as an input to DSSW [23].

Another specific research opportunity that may be able to exploit some of these technologies is associated with agent-based modeling of mass gatherings. The specific technologies referenced above are ideally suited to many mass gatherings such as the Olympics or the Hajj. The Hajj is the largest mass pilgrimage in the world, in which an estimated two-to-three million people participated in 2007. In addition to a large number of people in high density, physical conditions are difficult and thus it presents an opportunity for a large-scale disease such as influenza to take hold. Participants then disperse to their homes, mainly via public transport, and could easily influence the spread and outbreak of the disease. We anticipate cellular tracking to be a most effective means of acquiring modeling data, as well as pre-Hajj assembly and post-Hajj dispersal, with agent-based modeling justifying its efficacy.

Agent-based models also allow for modeling the effectiveness of a city's preparedness planning. This is obviously a massive undertaking, but one in which our individual city model could be useful in providing planners with policies and some degree of expectation as to how goods and services could be provisioned in the event of a catastrophe. Simple investigations as to how long food supplies would last and could be distributed will be modeled. Provisioning of resources extempore will lead to an aggravated and worsening disaster. As such, models like ours will contribute to preparedness planning. This can become an effective modeling tool for any city or municipality, allowing for provisioning not only of food and supplies but for inoculation services as well as temporary hospital and/or mortuary facilities. A detailed agent-based model offers the flexibility to undertake preparedness and mitigation-strategy exploration. It should be apparent that these models will require considerably different inputs of a non-obvious nature.

Epstein *et al. *[24] discuss the economic impact of restricting air travel as a policy in controlling a flu pandemic. Some of the paper is devoted to the study of interventions, such as travel restrictions which may affect the spread of a disease in either direction. The important point is that they are using their model and seeing how policy affects it, which is how we also see the DSSW being used in preparedness planning. In this paper, they estimate the impact of travel restrictions needing to be extreme to be effective (95% restrictions). Their model treats each city as an individual node and models flight patterns between cities. As such, it may be suitable to sit on top of the DSSW simulation within a city.

**Figure 12:**
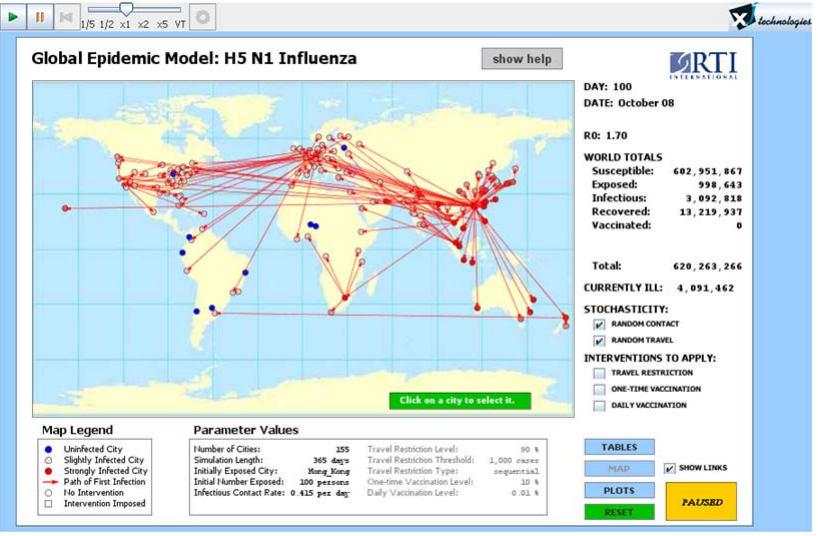
**Screenshot of global epidemic modeler **[26].

One of their big issues is the cost of implementing policy. They found that, unless the travel restrictions were extreme, the impact upon the spread of disease was negligible. This is not as an unexpected result, as the flight schedules and plans are clearly a highly connected network. The implication is immediate that for the graph to become disconnected, and thereby mitigate against disease spread, a large number of nodes or edges necessarily need to be removed. An extension or opportunity that may be of interest in pursuing is the use of *affinity propagation *[25] within a simulation to try and determine which flights to restrict as a policy aid. Figure 12 illustrates a screenshot of the simulation tool used in reference [24]. The economic impact of policy might be made somewhat easier if we look at the situation from an economist's perspective.

One final source of behavioral data worth considering is related to that of Google's recent "Flu Trends" [27]. Researchers "found that certain search terms are good indicators of flu activity. Google Flu Trends uses aggregated Google search data to estimate flu activity in your state up to two weeks faster than traditional systems" such as data collected by the CDC. The analysis of search terms is clearly an effective means of bio-surveillance. For the purposes of agent-based modeling tools such as DSSW, we are more concerned with data-mining opportunities that provide a means of collecting behavioral (spatial) patterns of movement that can be used as empirical inputs augmenting the simulation. Google mail (gmail) provides an example of this type. When using gmail, the web/mail server has a reasonable estimate of one's activity status (busy, available, idle, offline, etc.). In addition to status, one's web browser's IP address also provides coarse-grained information of where an individual is logged in. For example, during the day a student or professor would likely be using a machine on a department subnet of the University network. In the early morning and evening that person would be more typically using a machine in a home on an ISP's network and subnet. If gmail is accessed from a mobile device, the location is also known to various degrees. Some of this information needs to be inferred, some is made available when agreeing to use the service. In any event, considerably more sophisticated next-generation data mining will no doubt be able to provide at least coarse estimates of spatial behavior patterns extracted from similar web services such the email usage mentioned here. Eric Schmidt, CEO of Google, said, "From a technological perspective, it is the beginning" [29].

All of the data-mining opportunities outlined above also face the challenges of protecting privacy. There are a number of organizations such as the Electronic Privacy Information Center [28] who serve as watchdogs when privacy concerns arise. These organizations are very responsive as evidenced by their concern over Google's "Flu Trend" data [29] and their immediate action. It is also becoming apparent that our collective notions of privacy rights and needs are changing. We are aware of living in a social environment where increasingly, our personal information is already available to institutions (e.g. financial institutions, governments, web services, loyalty programs and on-line social networks). In addition, we readily give up personal privacy in exchange for something considered valuable (access, information, convenience, etc.).

### Opportunities for modeling HIV/AIDS

The DSSW agent-based simulator may be useful in helping to predict the spread of HIV/AIDS, as it places emphasis on patterns of behavior and data mining. However, without knowledge (albeit sparse) of patterns of behavior among different social and economic demographics relevant to HIV exposure, much of our simulation would be pure conjecture. This is in contrast to diseases such as influenza which really have no predisposed target group. Influenza is more "who"-agnostic than HIV. Thus, the variability in the probability of "catching a cold" among a population is much less pronounced as would be the case for HIV/AIDS.

In [31], risk networks in an agent-based modeling framework are used to simulate the spread of HIV. Preliminary results indicate that the knowledge of the actual network structure is of critical importance for the sexual transmission and of less importance for injection-related transmission. This type of result is of interest, as it illustrates that agent-based modeling can be effective even for diseases such as HIV/AIDS.

In this work, we consider the suitability of our DSSW agent-based model applied to HIV/AIDS proliferation. There are several factors which can be incorporated in a DSSW agent-based model for HIV/AIDS. Many factors are behavioral and social in nature, while others are founded on logistics, geography and socio-economics.

Demographics that are made available from disparate sources (which contain relevant factors) may very well be conducive to the modeling and simulation of HIV/AIDS in an urban setting. For instance, it is possible that meaningful patterns of behavior among different demographics can be extracted with conventional data-mining technologies. In this way, it would be possible to model individuals or groups of people living with HIV/AIDS (or are at higher risk of contracting the disease) among urban geographic clusters. Moreover, it may also be possible to assign risk to those already infected of further spreading the disease among their demographic. These are the kinds of informational dynamics which we conjecture to be useful in the composition of overall epidemiological agent-based models for HIV/AIDS. Ultimately, it is hoped that these models will be useful in circumventing the escalation of the disease among those demographics susceptible to a HIV/AIDS outbreak.

### Epidemiology in the Bronx: a blueprint for modeling HIV/AIDS

The borough of the Bronx in New York City has among the largest AIDS-related death rate per capita in the United States (10 times the national average [33]) that some are declaring are of "epidemic proportions". At present, there are more than 6,751 individuals known to be living with HIV and AIDS in the Bronx [34]. A massive undertaking is now underway to control the spread of the disease and prevent AIDS-related deaths by the voluntary testing of every single individual in the Bronx by 2011. This amounts to the ambitious testing of more than 250,000 individuals between the ages of 18 and 64.

In order to achieve the required goal, there are many HIV testing centers that have been established throughout the Bronx (about 40) with confidential name-based HIV/AIDS case reporting. Geographically, many testing centers are located near or within high HIV-risk areas which include churches, universities, health clinics, community centers, emergency rooms and doctors' offices [35]. Knowledge of these high HIV-risk-testing-centre locations is useful in the assignment of a DSSW schedule since they represent a "where" in the HIV/AIDS disease-spread model. This is important, since location is correlated with other factors to be incorporated in the model, which are in nature behavioral, social and socio-economically based.

The HIV epidemic leading to AIDS-related deaths in the Bronx is intimately linked to several of these factors. Because Bronx residents are poorer (poverty factor) and less educated (social inequality factor) than national standards, they often do not find out that they are HIV infected until late stages of the disease, when it becomes impossible or difficult to treat infection, which later develops into AIDS and results in death [35]. Particularly hard hit in the Bronx for HIV infection are the African American and Hispanic populations (poverty, social inequality, racial and cultural factors).

Other factors leading to a high rate of HIV infections among the more than 850,000 Bronx residents involve substance abuse and addiction. Injection drug use is a major risk factor for HIV. Alcohol and substance abuse are strongly linked to sexual risk (sexual promiscuity factor), which increases the likelihood of HIV infection as well [36]. Most at risk for HIV infection in the Bronx are males having unprotected sexual encounters with other males (MSM sexual encounter factor). This is most pronounced among young boys and men, particularly among ethnic minorities [37].

Needle exchange programs are known to have a significant benefit in reducing the risk of HIV contraction [38] (including other diseases such as hepatitis) among injection drug users and the general populous. Such programs offer free syringes to injection drug users in exchange for their used syringes. The concept is to enable the safe disposal of used syringes to preclude the re-use of potentially infected/contaminated syringes, hence reducing the possibility of spreading the infection to others. It has been reported that injection drug use directly or indirectly accounted for more than 36% of AIDS cases. In general, it is believed that needle exchange programs could lead to a 30% or greater reduction in HIV [39].

The Bronx has established several community-based needle exchange initiatives and offers several services (e.g., CitiWide Harm Reduction, New York Harm Reduction Educators, St. Ann's Corner of Harm Reduction) to address the unwanted re-use or public discarding of contaminated needles. CitiWide has revealed that more than 80% of their clientele that use their needle exchange services are HIV-positive.

There are many fixed-site and mobile-site locations in the Bronx for the collection and distribution of syringes. These locations are predetermined (where) and operate according to schedules (when). Both location and schedule information are publicly available. Such information can be data mined and incorporated into the DSSW agent-based model to track behavior patterns (clusters) of IV drug users and their relative interaction among others - some which may be at higher risk of HIV infection.

Furthermore, from a technical standpoint, it is feasible to attach RFID tags on every outbound syringe in order to track their progression through the needle exchange chain of distribution (i.e., when and where the syringe was handed out, to when and where the contaminated syringe came back). This information can also be assimilated in the DSSW agent-based model. For those drug users who simply choose to discard their contaminated syringes in public places, an RFID reader (used much like a metal detector) can be used to locate the RFID tagged syringes (even when hidden from view), making them much easier for one to locate for proper and safe disposal. It should be noted that the needles would be tracked with a secure ID/database, whereas individuals themselves are not tracked. The RFID tags on the needles are used to identify the needles upon return and to find them among the injection sites, on the streets (even in grass and clutter where they have been discarded). The purpose of this is twofold: to track the actual return rates (on a per usage basis, to evaluate the needle exchange program efficacy); and to locate the contaminated needles and collect them to prevent the general public (or "needle retrievers") from accidentally pricking oneself with an HIV/Hepatitis contaminated needle. A safe needle collection method will also prevent other IV drug users from using contaminated needles they find on the street. Locating needles is a major problem even in cities with needle exchange programs. Identification and invasion of privacy/anonymity (and the compromise thereof) are very low risk compared to the dangers associated with discarded needles.

Needles tagged and programmed with RFID identifiers/codes are only meaningful to the needle exchange depot itself (where they are programmed, interpreted and stored on a database). If the database is stolen, or seized by the authorities, it cannot be used to trace a drug-user's name or personal identification.

As an extreme measure to monitor the spread and progression of HIV/AIDS, controversial state Bills are being legislated in some countries (e.g., Indonesia) requiring some HIV/AIDS patients to be implanted with RFID microchips [40]. This would allow authorities to identify, track and ultimately punish those who deliberately infect others. Targeted are sex trade workers and IV drug users - in particular those of whom are somehow deemed to be at high risk for criminal conduct (negligence) in the deliberate (or reckless) infection of others. The human RFID "tagging" of HIV/AIDS infected persons for identification and tracking is controversial on several levels and understandably health workers and rights activists sharply criticized the plan.

While we have not discussed the feedback loop between DSSW agent-based modeling and such goals, which would in effect be changes in agents' schedules, this particular proposal brings the issue to the fore. The tagging initiative could potentially represent a means of augmenting an agent-based simulator with empirical inputs, insofar as to provide additional behavioural pattern resolution that would not otherwise be possible. However, human rights and privacy concerns would need to be carefully considered first.

Overall, there are statistically significant demographics which are currently being collected as a result of the widescale HIV/AIDS testing program along with needle exchange program initiatives. As a result, the Bronx in New York City could represent an ideal testbed for which to study and assess the efficacy of epidemiological modeling for HIV/AIDS, including the DSSW agent-based model we are considering.

### Modeling treatment modalities: controlling the spread of HIV/AIDS

The widespread usage of anti-viral and anti-retroviral medications for HIV has a significant effect on the prolongation and quality of life for those living with HIV/AIDS and may even have an effect on the spread of HIV disease (and the modeling therein). As such, it should be noted that many worldwide patients of HIV are now receiving antiretroviral therapy. This medication treatment modality appears to reduce the progress of HIV infection (in the very least, with respect to the prevention of mother-to-child transmission [32]) and may therefore have an impact on the overall spread of disease model.

Cautiously optimistic *immunotherapy *treatments for AIDS virus infections may also have a significant impact on the spread-of-disease model. The goal of such therapeutic vaccines is to modulate the course of disease by preventing suffering and prolonging life. The approach described in [41], while preliminary, offers new hope of this possibility.

Now under consideration as a potential new treatment modality for HIV/AIDS, *Nanomedicines *- which are currently under development - may have a significant impact in the treatment and progression of the disease. One such nanomedicine compound uses nano-particles of gold in combination with previously "failed" HIV drugs to rekindle the drug's ability to stop the virus from invading the body's immune system and gaining a cellular foothold [42]. Such nano-engineered drugs have the promise to offer a viable means for keeping the disease at bay by inhibiting the destructive nature of HIV/AIDS in an infected individual and to perhaps reduce their capacity to infect others, hence affecting the spread-of-disease model.

## Summary

Effectively, we are combining agent-based modeling with a heavy emphasis on data mining for the topology (the network) and individual agent's behavior. Neither of these concepts are new, but their combination is timely for epidemic modeling. The mining of data is now more widely accessible than ever, largely due in part to the proliferation of the internet and map utilities. We focused on opportunities that perhaps were initially intended for other purposes but appear timely in the context of attempting to develop the best possible model. The kinds of epidemics we are primarily concerned with are "who' agnostic, where there is no predisposition on the part of the virus as to who it infects. While we were motivated in this work to adapt our simulator to diseases such as HIV/AIDS, that problem is considerably more difficult to model as it involves greater complexity of behavior on the part of the agent. Part two highlights a list of limitations, extensions and research opportunities and provides a sampling of technologies that may have benefit in extracting or mining behavior from somewhat non-obvious or disparate sources. The final section of the paper outlines an approach where an agent-based model such as DSSW could be used to model a limited and constrained HIV/AIDS epidemic or outbreak. Through this paper we also hope we have brought opportunities and technologies to the attention of epidemic modelers that are perhaps more closely aligned to an engineer. Conversely, we hope we have brought opportunities in epidemic modeling to the attention of engineers.

## Competing interests

The authors declare that they have no competing interests.

## Authors' contributions

MB developed and implemented the agent based model for disease spread within an urban area. RDM developed the agent based model and wrote the manuscript. BWP provided background research on extending the agent based model of disease spread to HIV/AIDS and wrote parts of the manuscript.
